# Exploring the modulatory effects of spotted pumpkin (*Lagenaria breviflora*) fruit aqueous extract on growth performance, behaviour and blood profile of broiler chickens

**DOI:** 10.1093/tas/txag075

**Published:** 2026-06-30

**Authors:** Adebayo Oluwatomiwa Enitan, Ibiyemi Omolola Opowoye, Adebayo Vincent Jegede, Oyegunle Emmanuel Oke, Oluwagbemiga Olanrewaju Adeleye

**Affiliations:** Department of Animal Production and Health, Federal University of Agriculture, Abeokuta, Ogun State, 110001, Nigeria; Department of Animal Production and Health, Federal University of Agriculture, Abeokuta, Ogun State, 110001, Nigeria; Department of Animal Nutrition, Federal University of Agriculture, Abeokuta, Ogun State, 110001, Nigeria; Department of Animal Physiology, Federal University of Agriculture, Abeokuta, Ogun State, 110001, Nigeria; Department of Animal Production and Health, Federal University of Agriculture, Abeokuta, Ogun State, 110001, Nigeria

**Keywords:** antioxidant status, broiler chickens, growth performance, *Lagenaria breviflora*, phytogenic additive, serum biochemistry

## Abstract

Growing concerns over health risks and microbial resistance associated with synthetic growth promoters have increased interest in natural alternatives in poultry production. This study evaluated the effects of *Lagenaria breviflora* (spotted pumpkin) fruit aqueous extract on growth performance, behaviour, haematology, and serum biochemistry of broiler chickens. A total of 240 day-old Ross 308 chicks were assigned to six treatment groups in a 2 (300 and 500 g/L) × 3 (0, 3, and 4 times/week) factorial arrangement and administered the extract via drinking water from day 7 to 42. Growth performance indices included weight gain, feed intake, water intake, and feed conversion ratio (FCR). Behaviour was monitored using closed-circuit television, and blood parameters were determined using standard laboratory procedures. While the extract did not improve growth performance, it influenced behavioural activity and selected physiological indices. Birds receiving 300 g/L showed higher feed and water intake than those on 500 g/L. Administration frequency significantly (*P* < 0.05) affected growth, with the control group (0 time/week) recording the highest final weight and weight gain. Behaviour did not differ at the starter phase, but at the finisher phase, birds on 300 g/L exhibited greater feeding, drinking, walking, dust bathing, and wing-flapping activities than those on 500 g/L. Frequency of administration also influenced behavioural expression, with control and 3 times/week groups generally more active than birds treated 4 times/week. Haematological indices were not affected by treatment. Serum biochemical parameters indicated improved antioxidant status at higher concentration (500 g/L), as evidenced by reduced (*P* < 0.05) malondialdehyde (MDA) levels. Birds receiving 500 g/L had higher serum protein and albumin and lower MDA levels, indicating improved antioxidant status. These findings suggest that *Lagenaria breviflora* extract may support behavioural expression and antioxidant status without improving growth performance. Therefore, its application should be considered cautiously, and further studies are required before definitive recommendations can be made.

## Teaser

Spotted pumpkin (*Lagenaria breviflora*) fruit aqueous extract shows promise as a natural alternative to synthetic growth promoters in broiler production, enhancing behavioural activity and improving antioxidant status without adverse health effects.

## Introduction

Poultry makes a substantial contribution to food security and nutrition, providing energy, protein, and essential micronutrients to humans (Mottet and Tempio 2017; Kpomasse et al. 2021; Onagbesan et al. 2023). In addition, poultry production is characterized by rapid growth rates and efficient feed conversion, making it a key component of global livestock systems (Kpomasse et al. 2023; Ajayi et al. 2022; Nassar 2026; Osei-Akoto et al. 2026). Poultry meat and egg production have increased considerably worldwide over the past 50 years. Poultry farming represents a critical sector in the global agro-industry, providing a sustainable source of protein for an ever-growing human population. To cope with market demand for protein (meat), modern broilers are reaching market age more quickly each year (Kleyn and Chrystal 2008;  Uyanga et al. 2023).

Antibiotics have been used for many decades in the poultry industry to enhance production, promote growth performance, and protect birds from pathogenic microbes (Paintsil et al. 2021). Enhanced poultry performance is attributed to improved appetite and feed conversion efficiency, stimulation of the immune system, increased vitality, and regulation of the intestinal microflora (Peric et al. 2009). Broadly, antibiotics are used in phytosanitary treatments, feed additives, and prophylactic treatments in animals and humans. Despite these important roles, improper use of antibiotics in animal farming has been reported to increase antimicrobial-resistant bacteria as a public health threat (Nhung et al. 2017; Oniciuc et al. 2018), residues in animal products, and cause environmental pollution (Carvalho and Santos 2016; Gonzalez and Angeles 2016). With the elimination of antibiotic growth promoters in food animal production and policies and approaches in several countries in response to the growing global threat of antibiotic resistance, alternatives are urgently needed to prevent disease and promote growth. Several alternative approaches and products that do not exert selection pressure for the development of antibiotic resistance have been studied and developed over the last decade (Rahman et al. 2022).

The increasing restrictions on antibiotic growth promoters have led to the exploration of plant-derived feed additives such as basil, oregano, thyme, sage, and parsley, which have demonstrated antimicrobial, antioxidant, and growth-promoting properties in poultry (Abd El-Hack et al. 2022; Adjei-Mensah et al. 2022; Akosile et al. 2023; Vlaicu et al. 2023; Ashour et al. 2025; Cojocariu et al. 2026). Compared with synthetic antibiotics or inorganic chemicals, phytogenic compounds are considered natural and residue-free and are considered ideal feed additives in food animal production (Hashemi et al. 2008; Akosile et al. 2023; Oni et al. 2024). Among these alternatives, Phytogenic feed additives (PFAs), derived from plant materials, are gaining attention due to their potential to enhance productivity and health without contributing to antimicrobial resistance (Abd El-Ghany 2020; Tokofai et al. 2020, 2021; Oke et al. 2021). The antimicrobial, appetising, and digestive stimulant properties of these compounds further enhance their value in poultry nutrition (Diniz et al. 2020). The exploration of new medicinal plant-derived products has become essential to increasing broiler production. In Nigeria, numerous herbs and plant components, including leaf meals, extracts, seeds, fruits, and tree barks, have been utilised in various studies as alternatives to standard feeds, feed ingredients, growth enhancers, and antibiotics. In addition to the use of these plants, research by Ekunseitan et al. (2017), Nworgu et al. (2018), and has utilised *Lagenaria breviflora* fruit as an alternative to antibiotics in animal production, particularly poultry. *Lagenaria breviflora*, commonly known as spotted pumpkin or wild bottle gourd, is a member of the Cucurbitaceae family. The bioactive components of *Lagenaria breviflora*, such as alkaloids, flavonoids, saponins, and tannins, contribute to its antimicrobial, anti-inflammatory, and antioxidant effects (Adeyemo et al. 2015; Adedosu et al. 2019).

Extracts of *Lagenaria breviflora* have been used in various studies and have shown improvements in bird health (Ekunseitan et al. 2017; Adeyemo et al. 2024). In laying hens, the extract had no deleterious effect on bird health when raised on modified housing systems (Ekunseitan et al. 2017). Egbeyale et al. (2021) reported that supplementation of *Lagenaria breviflora* fruit extract at 75 g/L improved water intake at the starter phase and feed-to-gain ratio at the finisher phase of broiler chickens. These various uses of *Lagenaria breviflora* fruit extract in poultry demonstrate its positive effects on growth parameters and health indicators and provide insights into its potential as a natural feed additive. Despite increasing interest in phytogenic feed additives, limited information exists on the combined effects of dosage and administration frequency of *Lagenaria breviflora* extract delivered via drinking water, particularly regarding behavioural responses and physiological adaptation in broiler chickens. Therefore, this study provides an integrative evaluation of growth performance, behavioural responses, and physiological indices using a factorial dosing-frequency approach.

## Materials and methods

### Animal care

All experimental procedures were conducted in accordance with the ethical standards for animal experimentation and approved by the Research Ethics Committee of the Federal University of Agriculture, Abeokuta, Nigeria.

### Experimental site

The research was carried out at the Poultry Unit of the Directorate of University Farm (DUFARMS), Federal University of Agriculture, Abeokuta (FUNAAB), Ogun State, Nigeria. The site is located in the rainforest zone of South-west Nigeria, at latitude 7°13′N, longitude 3°26′E, and an Altitude of 98 m above sea level. The climate is humid, with a mean annual precipitation of 1003 mm. The annual mean temperature and humidity are 34.79% and 82%, respectively.

### Sourcing and preparation of test ingredient

Spotted pumpkin (*Lagenaria breviflora*) fruits were purchased from a local market situated in Abeokuta, Ogun State. They were weighed according to the intended concentrations to be prepared: 300 g/L and 500 g/L in water. After weighing, the fruits were cut into smaller pieces and placed in two separate drums. The drums were filled with water in accordance with the weight of the ingredients cut. The preparation method used in this study is more accurately described as an aqueous macerate rather than a standardized extract. The fruits were soaked in water at a ratio of 300 g/L and 500 g/L at ambient temperature (27–30°C) for for 72 hours, after which the pulp was removed and discarded. The mixture was sieved to remove solid residues before administration. There was no chemical standardization of bioactive compounds performed, and this is acknowledged as a limitation. The sieved water was used as drinking water for the birds throughout the experimental period.

### Experimental birds and management

A total of 240 unsexed day-old broiler chicks (Ross 308 strain) were purchased for the study. The birds were sourced from a reputable hatchery in Ibadan, Oyo State. Before the arrival of the chicks, the pen and surrounding environment were thoroughly cleaned, disinfected and fumigated, then allowed to rest for two weeks. All equipment was also properly sanitised before use. Before the birds were brought into the farm, wood shavings were spread on the floor of the brooding pen. The entire brooding pen was measured and divided into six units, each representing a treatment. The brooding pen was preheated with coal pots and electric bulbs to provide warmth for the birds.

The birds were reared in an open-sided poultry house on a deep-litter system, with wood shavings used as bedding at an approximate depth of 5–8 cm. The house was naturally ventilated through open sides fitted with wire mesh, allowing free air movement while preventing predator entry. The facility was divided into six pens corresponding to the experimental treatments.

Upon arrival, the birds were weighed and randomly assigned to 6 treatments, with 40 birds per treatment. An approximate stocking density of 10 birds/m^2^ was used. The birds were given commercial feed and drinking water ad libitum throughout the experiment. A starter diet containing 2800 kcal/kgME and 22% CP was given for the first four (4) weeks, and a finisher diet containing 3000 kcal/kgME and 18% CP was given for the next two weeks. The temperature and humidity of the brooding pen were monitored using a thermo-hygrometer mounted on the pen wall. Administration of the spotted pumpkin fruit extract commenced on day seven (7) till the end of the experiment.

### Experimental design

The experiment used a 2 × 3 factorial arrangement with two concentrations (300 and 500 g/L) and three administration frequencies (0, 3, and 4 times/week). A separate control group was used for each of the concentrations (0 times/week, no extract) for comparison. Two hundred and forty birds (240) were randomly assigned to the six treatments, with four replicates of 10 birds per replicate.

The experimental layout was as follows;

Treatment 1: The birds in this treatment were the control group for the concentration 300 g/L *Lagenaria breviflora* fruit aqueous extract.

Treatment 2: The birds in this treatment were administered 300 g/L *Lagenaria breviflora* fruit aqueous extract as drinking water. Three times a week throughout the experiment.

Treatment 3: The birds in this treatment were administered 300 g/L *Lagenaria breviflora* fruit aqueous extract as drinking water. Four times a week throughout the experiment.

Treatment 4: The birds in this treatment were the control group for the concentration of 500 g/L *Lagenaria breviflora* fruit aqueous extract.

Treatment 5: The birds in this treatment were administered 500 g/L *Lagenaria breviflora* fruit aqueous extract as drinking water. Three times a week throughout the experiment.

Treatment 6: The birds in this treatment were administered 500 g/L *Lagenaria breviflora* fruit aqueous extract as drinking water. Four times a week throughout the experiment.

### Data collection

#### Evaluation of growth performance

Growth parameters were evaluated weekly for each replicate in terms of body weight gain (BWG), feed intake (FI), water intake, and feed conversion ratio (FCR).

##### Body weight gain

Birds were weighed weekly to monitor weight gain. The weight gain was calculated as:


Total Weight Gain (g) = Final Body Weight (g)-Initial Body Weight (g).

##### Feed intake

Feed intake was measured on a replicate pen basis, which served as the experimental unit. A known quantity of feed was offered daily to each pen, and the residual feed was collected and weighed the following day. Feed intake per pen was calculated as the difference between feed offered and feed refused, and values were expressed on a per bird basis by dividing by the number of birds in each replicate. Each replicate pen contained 10 birds and was provided with one feeder, ensuring equal access to feed. Feed was supplied ad libitum throughout the experimental period. Feed intake was calculated using the formula:


Feed intake (g)=Feed given (g)−Feed leftover (g)

##### Water intake

Water intake was measured on a replicate pen basis, which served as the experimental unit. A known volume of water was provided daily to each pen using graduated drinkers, and the remaining water was measured after 24 hours. Water intake per pen was calculated as the difference between the volume of water offered and the volume remaining, and values were expressed on a per bird basis by dividing by the number of birds in each replicate.

Each replicate pen contained 10 birds and was provided with one drinker, ensuring equal access to water. Water was supplied ad libitum throughout the experimental period. Care was taken to minimise spillage, and any observed spillage was accounted for during measurement. The calculation was as follows:


Water intake(l)=Water given(l)−Water left over(l)

##### Feed conversion ratio (FCR)

This is the ratio of feed intake and their subsequent body weight gain:


$$\mathrm{FCR}=\frac{\mathrm{Feed}\;\mathrm{intake}\;(g)}{\mathrm{Body}\;\mathrm{Weight}\;\mathrm{gain}\;(g)}$$

#### Behavioural parameters

Behaviour was assessed using continuous scan sampling at defined intervals from recorded 8 unit closed-circuit Television (CCTV) footage. Each observation session lasted 8 hours. The birds were observed after the brooding phase, using specific behavioural parameters: feeding, drinking, dustbathing, walking, and wing-flapping. Six-CCTV cameras were installed to record behavioural activities of birds for six (6) hours thrice weekly, at weeks 4, 5 and 6. The captured videos were reviewed to extract data on the behavioural activities of birds by quantifying the frequency with which the target behaviour was observed.

#### Determination of haematological parameters

In the sixth week of the experiment, three birds were randomly selected from each replicate for blood sampling. Blood samples were collected in the early hours of the day (6:00 am). Blood samples were collected via the wing veins into ethylenediaminetetraacetic acid (EDTA) bottles for haematological evaluation. Blood samples were collected in bottles using sterile syringes and needles and transported to the laboratory for analysis. Immediately after collection, bottles were placed in an ice pack and transported to the laboratory. Haemoglobin concentration was estimated using the cyanmethemoglobin method: 0.02 ml of blood was mixed with 5 ml of Drabkin’s Solution. The mixture was allowed to settle for 10 minutes to facilitate colour development, and the absorbance was measured using a spectrophotometer. Packed Cell Volume (PCV) was measured by centrifuging each blood sample in a heparinised capillary tube for 5 minutes in a hematocrit centrifuge, and the results were read using a hematocrit reader. The White Blood Cell (WBC) count was performed using the Neubauer Chamber Method, in which the blood sample was diluted 1:20 with a white cell diluent. The Red Blood Cell (RBC) count was determined by diluting the sample 1:200 with a red blood cell diluent. Cell counts were performed for each sample. While observing the samples under a microscope, the percentages of various cell types (i.e., lymphocytes, eosinophils, basophils, heterophils, and monocytes) were calculated from cell counts. Standard equations were used to determine the red blood cell indices, including mean corpuscular volume (MCV), mean corpuscular haemoglobin (MCH), and mean corpuscular haemoglobin concentration (MCHC).


$$\begin{array}{c} MCV\;=\;\frac{\mathrm{Packed}\;\mathrm{cell}\;\mathrm{volume}}{\mathrm{Red}\;\mathrm{blood}\;\mathrm{cell}}X\;10\\ \begin{array}{c} MCH\;=\;\frac{\mathrm{Haemoglobin}}{\mathrm{Red}\;\mathrm{blood}\;\mathrm{cell}}X\;10\\ M\mathrm{CHC}\;=\;\frac{\mathrm{Haemoglobin}}{\mathrm{Packed}\;\mathrm{cell}\;\mathrm{volume}}\;X\;100 \end{array} \end{array}$$

#### Determination of serum biochemical indices

Data were collected at the sixth week of the experiment. Blood was collected from a total of 72 experimental birds; three (3) from each replicate, and 12 per treatment group through the jugular vein, which is located at the neck of the animal. Blood was collected into labelled plain sample bottles and then transported to the laboratory for serum analysis.

Commercial diagnostic kits (Randox Laboratories, U.K.) were used, and analyses were performed in duplicate using a spectrophotometer. Serum protein levels were assessed using the Biuret method. A mixture of the reagent and serum sample was incubated for 30 minutes at a temperature of 20–25°C, and the absorbance of both the sample and the standard was measured against a blank at a wavelength of 540 nm. Serum albumin concentration was determined, while globulin concentration was calculated as the difference between total protein and albumin levels. Serum glucose was measured calorimetrically using the glucose oxidase/phenol and 4-aminoantipyrine method, which involves the enzyme glucose oxidase and peroxidase (NCCLS, 1990). Alanine and aspartate aminotransferase levels were measured using a spectrophotometer, ilewith a commercial Randox kit.

#### Statistical analysis

*Lagenaria breviflora* Data were analysed using a two-way analysis of variance (ANOVA) within the General Linear Model procedure of Minitab (2018), considering the main effects of extract concentration (300 and 500 g/L), administration frequency (0, 3, and 4 times/week), and their interaction. The replicate pen (10 birds per replicate) served as the experimental unit for all performance and behavioural data, while individual birds were considered the experimental unit for haematological and serum biochemical analyses. Prior to analysis, data were tested for normality using the Shapiro–Wilk test and for homogeneity of variance using Levene’s test. All data met the ANOVA assumptions. Where significant differences were detected, means were separated using Tukey’s Honestly Significant Difference (HSD) test. Statistical significance was declared at *p* < 0.05.

## Results

### Main effects of concentration and frequency of administration of *Lagenaria breviflora* fruit aqueous extract on the growth performance of broiler chickens

Table 1 shows the main effects of concentration and frequency of administration of *Lagenaria breviflora* fruit aqueous extract on the growth performance of broiler chickens. For the effect of concentration on performance parameters, birds on 300 g/L *Lagenaria breviflora* fruit aqueous extract had the highest (*p* < 0.05) water intake (8480.00 ml) and feed intake (3793.50 g), while those on 500 g/L *Lagenaria breviflora* fruit aqueous extract had the lowest values (*p* < 0.05).

**Table 1:** Main effects of concentration and frequency of administration of *Lagenaria breviflora* fruit aqueous extract on the growth performance of broiler chickens.

	Concentration				Frequency of administration				
Parameters	300 g/L	500 g/L	SEM	*P*-value	0 time/week	3 times/week	4 times/week	SEM	*P*-value
***Initial weight (g/bird)***	285.87	281.15	1.68	0.062	282.05	283.02	285.45	2.05	0.497
***Final weight (g/bird)***	1937.40	1905.40	30.20	0.464	2052.30^a^	1854.40^b^	1854.40^b^	37.00	0.002
***Weight gain (g/bird)***	1651.50	1624.30	30.60	0.537	1770.20^a^	1574.50^b^	1568.90^b^	37.40	0.002
***Feed intake (g/bird)***	3793.50^a^	3705.67^b^	1.46	0.000	3703.75^a^	3689.25^b^	3655.75^c^	1.78	0.000
***Water intake (ml/bird)***	8480.00^a^	8174.50^b^	1.39	0.000	9183.38^a^	8484.63^b^	8135.75^c^	1.70	0.000
***Feed conversion ratio***	2.31	2.29	0.04	0.821	2.21	2.34	2.34	0.05	0.174

abc Means in a row with different superscripts are significantly different (*p* < 0.05).

SEM: Standard error of mean.

Significantly (*p* < 0.05) different final weight and weight gain were observed in birds due to the effect of the frequency of administration of *Lageneria breviflora* fruit aqueous extract at different times. The final weight (2052.30 g), weight gain (1770.20 g), feed intake (3703.75 g), and water intake (9183.38 ml) of broiler chickens at the 0 time/week frequency were highest (*p* < 0.05) compared with those in other treatment groups. While final weight and weight gain showed similar values, feed and water intake in birds of 3 and 4 times/week frequencies were significantly different and lowest (*p* < 0.05) at the 4 times/week treatment group.

### Interactive effects of concentration and frequency of administration of Lagenaria breviflora fruit aqueous extract on the growth performance of broiler chickens

The interactive effects of concentration and frequency of administration of *Lagenaria breviflora* fruit aqueous extract are presented in Table 2. Of all the parameters measured, only feed and water intake were significantly affected (*p* < 0.05).

**Table 2:** Interactive effects of concentration and frequency of administration of *Lagenaria breviflora* fruit aqueous extract on the growth performance of broiler chickens.

	**300 g/L *Lagenaria breviflora***			**500 g/L *Lagenaria breviflora***				
Parameters	0 time/week	3 times/week	4 times/week	0 time/week	3 times/week	4 times/week	SEM	*P*-value
***Initial weight (g/bird)***	283.75	287.55	286.30	280.35	278.50	284.60	2.91	0.062
***Final weight (g/bird)***	2023.30	1910.00	1878.80	2081.30	1805.00	1830.00	52.30	0.310
***Weight gain (g/bird)***	1739.60	1622.50	1592.50	1800.90	1526.50	1545.40	52.90	0.337
***Feed intake (g/bird)***	3861.00^b^	3748.75^d^	3770.75^c^	3946.50^a^	3629.75^e^	3540.75^f^	2.52	0.000
***Water intake (ml/bird)***	9296.00^a^	8527.00^c^	8320.50^e^	9070.75^b^	8442.25^d^	7951.00^f^	2.41	0.000
***FCR***	2.23	2.31	2.38	2.20	2.38	2.30	0.008	0.593

a,b Means in a row with different superscripts are significantly different (*p* < 0.05).

SEM: Standard error of mean; FCR: Feed Conversion Ratio.

Birds in the treatments group administered 500 g/L of *Lagenaria breviflora* fruit aqueous extract at 0 time/week had the highest (*p* < 0.05) feed intake (3946.50 g), while those on administration of 500 g/L *Lagenaria breviflora* fruit aqueous extract at 4 times/week had the least (*p* < 0.05) feed intake. Birds in the treatment group administered 300 g/L of *Lagenaria breviflora* fruit aqueous extract at 0 time/week had the highest (*p* < 0.05) water intake (9296.00 ml), while those on 500 g/L of *Lagenaria breviflora* fruit aqueous extract at 4 times/week had the least (*p* < 0.05).

### Main effects of concentration and frequency of administration of *Lageneria breviflora* fruit aqueous extract on the haematological parameters of broiler chickens

Table 3 shows the main effects of concentration and frequency of administration of *Lageneria breviflora* fruit aqueous extract on the haematological parameters of broiler chickens. All parameters measured were not significantly (*p* > 0.05) influenced by concentration and frequency of administration of *Lagenaria breviflora* fruit aqueous extract.

**Table 3:** Main effects of concentration and frequency of administration of *Lagenaria breviflora* fruit aqueous extract on the haematological parameters of broiler chickens.

	Concentration				Frequency of administration				
Parameters	300 g/L	500 g/L	SEM	*P*-value	0 time/week	3 times/week	4 times/week	SEM	*P*-value
***PCV (%)***	28.69	29.18	0.57	0.560	29.25	29.00	28.54	0.70	0.769
***Hb (g/dl)***	9.69	9.87	0.17	0.410	9.47	9.79	5.63	0.20	0.547
***RBC (x1012/L)***	2.45	2.56	0.05	0.103	2.50	2.57	2.46	0.06	0.015
***WBC (×109/L)***	15.23	15.53	0.26	0.406	14.96	15.93	15.24	0.31	0.086
***Neutrophil (%)***	28.47	29.17	0.42	0.244	29.29	29.04	28.13	0.51	0.244
***Lymphocytes (%)***	69.36	68.86	0.43	0.409	68.25	69.04	70.04	0.74	0.068
***Eosinophils (%)***	0.56	0.56	0.10	1.000	0.75	0.42	0.50	0.12	0.145
***Basophil (%)***	0.61	0.79	0.12	0.312	0.63	0.75	0.71	0.14	0.818
***Monocytes (%)***	0.97	0.67	0.12	0.079	1.04	0.79	0.63	0.15	0.143
***MCV (fl)***	117.78	114.80	2.06	0.308	119.10	113.58	116.18	2.52	0.308
***MCH (pg)***	39.85	39.98	0.68	0.365	40.54	38.39	39.30	0.83	0.192
***MCHC (g/dl)***	33.86	33.97	0.20	0.715	34.05	33.83	33.86	0.24	0.793

SEM, Standard error of mean; PCV, Packed Cell Volume; RBC, Red Blood Cell; WBC, White Blood Cell; MCV, Mean Corpuscular Volume; MCH, Mean Corpuscular Haemoglobin; MCHC, Mean Corpuscular Haemoglobin Concentration.

### Interactive effects of concentration and frequency of administration of *Lagenaria breviflora* fruit aqueous extract on the haematological parameters of broiler chickens

The Interactive effects of concentration and frequency of administration of *Lagenaria breviflora* fruit aqueous extract on the haematological parameters of broiler chickens are shown in Table 4. None of the measured parameters were significantly affected (*p* > 0.05).

**Table 4:** Interactive effects of concentration and frequency of administration of *Lagenaria breviflora* fruit aqueous extract on the haematological parameters of broiler chickens.

	**300 g/L *Lagenaria breviflora***			**500 g/L *Lagenaria breviflora***				
Parameters	0 time/week	3 times/week	4 times/week	0 time/week	3 times/week	4 times/week	SEM	*P*-value
***PCV (%)***	30.00	28.08	28.00	28.50	29.92	29.08	0.10	0.217
***Hb (g/dl)***	10.04	9.58	9.45	9.80	10.00	9.81	0.29	0.508
***RBC (x10^12/L^)***	2.56	2.40	2.38	2.40	2.74	2.54	0.28	0.511
***WBC (×10^9/L^)***	15.36	15.72	14.62	14.59	16.15	15.89	0.44	0.070
***Neutrophil (%)***	28.25	28.58	28.58	30.33	29.50	27.67	0.72	0.121
***Lymphocytes (%)***	69.08	69.42	69.58	67.42	68.67	70.50	0.74	0.213
***Eosinophils (%)***	0.75	0.33	0.58	0.75	0.50	0.42	017	0.634
***Basophil (%)***	0.67	0.67	0.50	0.58	0.83	0.92	0.20	0.464
***Monocytes (%)***	1.17	1.00	0.75	9.12	0.58	0.50	0.21	0.900
***MCV (fl)***	117.83	117.92	117.61	120.38	109.25	114.76	3.56	0.297
***MCH (pg)***	39.51	40.24	39.80	41.58	38.55	38.80	1.17	0.056
***MCHC (g/dl)***	33.503	34.17	33.92	34.60	33.50	33.81	0.35	0.059

SEM, Standard error of mean; PCV, Packed Cell Volume; RBC, Red Blood Cell; WBC, White Blood Cell; MCV, Mean Corpuscular Volume; MCH, Mean Corpuscular Haemoglobin; MCHC, Mean Corpuscular Haemoglobin Concentration.

### Main effects of concentration and frequency of administration of *Lagenaria breviflora* fruit aqueous extract on the serum biochemical indices of broiler chickens

Table 5 presents the main effects of concentration and frequency of administration of *Lagenaria breviflora* fruit aqueous extract on the serum biochemical indices of broiler chickens. All parameters measured were not significantly (*p* > 0.05) influenced by the concentration of *Lagenaria breviflora* fruit aqueous extract, except total protein, albumin, AST, HDL and MDA. Total protein increased with concentration. A similar trend was also observed in the values of albumin, AST and HDL. Chickens on administration of 500 g/L of *Lageneria breviflora* fruit aqueous extract had significantly higher (*p* < 0.05) total protein (52.31 g/L) and albumin levels (34.64 g/L) compared to those receiving 300 g/L (45.53 g/L and 31.03 g/L, respectively. Higher (*p* < 0.05) AST levels were observed in the 500 g/L group (41.36 U/L) compared to the 300 g/L group (36.49 U/L). Lower (*p* < 0.05) MDA levels were found in the 500 g/L group (2.96 μmol/L) compared to the 300 g/L group (3.37 μmol/L).

**Table 5:** Main effects of concentration and frequency of administration of *Lagenaria breviflora* fruit aqueous extract on the serum biochemical indices of broiler chickens.

	Concentration				frequency of administration				
Parameters	300 g/L	500 g/L	SEM	*P*-value	0 time/week	3 times/week	4 times/week	SEM	*P*-value
***Glucose (mg/dL)***	142.14	141.07	2.81	0.790	151.94^a^	138.61^b^	134.26^b^	3.45	0.005
***Total protein (g/L)***	45.53^b^	52.31^a^	1.01	0.000	54.35^a^	44.30^b^	48.11^ab^	1.24	0.000
***Albumin(g/L)***	31.03^b^	34.64^a^	0.79	0.005	39.70^a^	30.34^b^	28.47^b^	0.96	0.000
***Globulin (g/L)***	14.49	17.67	1.10	0.056	14.65^b^	13.96^b^	19.64^a^	1.35	0.016
***Albumin/globulin ratio***	2.22	2.27	0.17	0.864	2.80^a^	2.32^ab^	1.62^b^	0.21	0.003
***Urea (g/L)***	50.92	54.14	1.66	0.188	59.49^a^	47.78^b^	50.32^b^	2.04	0.002
***Creatinine (mg/dL)***	0.49	0.46	0.03	0.545	0.62^a^	0.38^b^	0.42^b^	0.04	0.002
***ALT (U/L)***	57.85	61.62	1.98	0.195	64.74	57.89	56.58	2.43	0.062
***AST (U/L)***	36.49^b^	41.36^a^	1.46	0.030	47.83^a^	35.20^b^	33.75^b^	1.79	0.000
***Triglyceride (mg/dL)***	86.98	89.20	2.47	0.534	93.28	88.07	82.91	3.03	0.080
***Cholesterol (mg/dL)***	116.79	118.63	2.52	0.612	124.82^a^	114.06^b^	114.24^b^	3.09	0.037
***HDL (mg/dL)***	51.00^b^	57.50^a^	2.03	0.037	48.59^b^	56.63^a^	57.53^a^	2.49	0.039
***VLDL (mg/dL)***	17.40	17.84	0.50	0.534	18.66	17.61	16.58	0.61	0.080
***LDL (mg/dL)***	48.39	43.29	3.74	0.349	57.57^a^	39.82^b^	40.13^b^	4.58	0.020
***MDA (μmol/L)***	3.37^a^	2.96^b^	0.11	0.016	4.35^a^	2.76^b^	2.39^b^	0.13	0.000

abc Means on the same row having different superscripts are significantly (*p* < 0.05) different.

SEM, Standard error of mean; AST, Aspartate aminotransferase; ALT, Alanine aminotransferase; MDA, Malondialdehyde; LDL, low density lipoprotein, VLDL, very low density lipoprotein, HDL, High density lipprotein, xx.

The frequency of administration of *Lagenaria breviflora* fruit aqueous extract significantly affected all measured parameters, except ALT, Triglyceride, and VLDL (*p* < 0.05). Chickens not receiving *Lagenaria breviflora* fruit aqueous extract (0 time/week) had higher (*p* < 0.05) glucose and Total protein levels (151.94 mg/dL) compared to those administered thrice (138.61 mg/dL) or four times a week (134.26 mg/dL). Higher albumin levels were observed in the 0 times/week group (39.70 g/L), while globulin levels were highest in the group that received *Lagenaria breviflora* fruit aqueous extract four times a week (19.64 g/L). The albumin/globulin ratio was similar at an administration frequency of 3 times/week, but differed with higher values at 0 times/week and lower values at 4 times/week. Lower urea, creatinine, and AST levels were observed in chickens administered aqueous extracts of *Lagenaria breviflora* fruit three or four times weekly. Cholesterol levels were higher in the non-administered group (124.82 mg/dL), while HDL levels were higher in chickens on administration of *Lagenaria breviflora* fruit aqueous extract 3 times (56.63 mg/dL) or 4 times/week (57.53 mg/dL). Significantly (*p* < 0.05) lower LDL and MDA levels were observed in chickens administered *L. breviflora* fruit aqueous extract 3times or 4timesweek compared to the non-administered group (4.35 μmol/L).

### Interactive effects of concentration and frequency of administration of *Lagenaria breviflora* fruit aqueous extract on the serum biochemical indices of broiler chickens

Table 6 presents the Interactive effects of concentration and frequency of administration of *Lagenaria breviflora* fruit aqueous extract on the serum biochemical indices of broiler chickens. Lowered total protein values were observed in birds on administration of 300 g/L of *Lagenaria breviflora* fruit aqueous extract at 3 and 4 times/week and those on administration of 500 g/L of *Lagenaria breviflora* fruit aqueous extract at 3 times/week. Significantly (*p* < 0.05) increased values were obtained in birds administered 500 g/L of *Lagenaria breviflora* fruit aqueous extract at 0 and 4 times/week and those on administration of 300 g/L of *Lagenaria breviflora* fruit aqueous extract at 0 times/week. Creatinine differed significantly, with the highest value at 500 g/L of extract at 0 times/week (*p* < 0.05), compared with the lowest value at 4 times/week (*p* < 0.05). MDA differed significantly (*p* < 0.05) with birds on oral administration of 300 g/L and 500 g/L of *Lagenaria breviflora* fruit aqueous extract at 0 times/week, having the highest (4.21 μmol/L, 4.50 μmol/L) values, while birds administered 500 g/L of *Lagenaria breviflora* fruit aqueous extract at 4 times/week had the lowest (2.00 μmol/L).

**Table 6:** Interactive effects of concentration and frequency of administration of *Lagenaria breviflora* fruit aqueous extract on the serum biochemical indices of broiler chickens.

	**300 g/L *Lagenaria breviflora***			**500 g/L *Lagenaria breviflora***				
Parameters	0 time/week	3 times/week	4 times/week	0 time/week	3 times/week	4 times/week	SEM	*P*-value
***Glucose (mg/dL)***	148.85	140.58	137.00	155.04	136.63	131.53	4.87	0.445
***Total protein (g/L)***	50.99^ab^	45.07^bc^	40.53^c^	57.71^a^	43.53b^c^	55.69^a^	1.75	0.001
***Albumin(g/L)***	36.65	29.64	26.81	42.75	31.03	30.13	1.36	0.249
***Globulin (g/L)***	14.33^b^	15.43^b^	13.72^b^	14.97^b^	12.49^b^	25.56^a^	1.90	0.003
***Albumin/globulin ratio***	2.60	2.10	1.98	2.10	2.54	1.27	0.29	0.105
***Urea (g/L)***	57.80	44.36	50.59	61.19	51.19	50.04	2.88	0.457
***Creatinine (mg/dL)***	0.59^ab^	0.35^bc^	0.53^abc^	0.66^a^	0.40^abc^	0.31^c^	0.06	0.046
***ALT (U/L)***	63.29	53.16	57.11	66.18	62.63	56.05	3.43	0.325
***AST (U/L)***	46.05	32.76	30.66	49.61	37.63	36.84	2.53	0.874
***Triglyceride (mg/dL)***	87.31	88.82	84.80	99.25	87.31	81.03	4.29	0.170
***Cholesterol (mg/dL)***	121.63	116.23	112.50	128.00	111.90	115.99	4.37	0.464
***HDL (mg/dL)***	48.39	51.61	53.01	48.80	61.65	62.05	3.52	0.344
***VLDL (mg/dL)***	17.46	17.64	16.96	19.85	17.46	16.21	0.86	0.170
***LDL (mg/dL)***	55.78	46.86	42.53	59.36	32.79	37.73	6.48	0.414
***MDA (μmol/L)***	4.21^a^	3.13^b^	2.77^bc^	4.50^a^	2.39 ^cd^	2.00^d^	0.188	0.017

abc Means on the same row having different superscripts are significantly (*p* < 0.05) different.

SEM, Standard error of mean; AST, Aspartate aminotransferase; ALT, Alanine aminotransferase; MDA, Malondialdehyde; LDL, low density lipoprotein, VLDL, very low density lipoprotein, HDL, High density lipoprotein.

### Main effects of concentration and frequency of administration of *Lagenaria breviflora* fruit aqueous extract on the behaviour of broiler chickens at the starter phase

The main effects of concentration and frequency of administration of *Lagenaria breviflora* fruit aqueous extract on the behaviour of broiler chickens at the starter phase are presented in Table 7. All parameters measured were not significantly (*p* > 0.05) influenced.

**Table 7:** Main effects of concentration and frequency of administration of *Lagenaria breviflora* fruit aqueous extract on the behaviour of broiler chickens at the starter phase.

	Concentration				Frequency of administration				
Parameters	300 g/L	500 g/L	SEM	*P*-value	0 time/week	3 times/week	4 times/week	SEM	*P*-value
***Feeding (freq.)***	19.21	20.01	0.70	0.435	19.38	18.48	20.96	0.86	0.158
***Drinking (freq.)***	20.32	18.56	1.20	0.320	17.89	20.94	19.50	1.47	0.370
***Dust bathing (freq.)***	0.91	0.89	0.11	0.890	0.74	0.78	0/98	0.14	0.375
***Wing flapping (freq.)***	7.29	5.39	0.74	0.095	5.05	5.60	8.38	0.91	0.051
***Walking (freq.)***	6.91	5.80	0.38	0.60	6.00	6.05	7.02	0.46	0.258

SEM, Standard error of mean.

### Interactive effects of concentration and frequency of administration of *Lagenaria breviflora* fruit aqueous extract on the behaviour of broiler chickens at the starter phase

Table 8 presents the interactive effects of concentration and frequency of administration of *Lagenaria breviflora* fruit aqueous extract on the behaviour of broiler chickens during the starter phase. None of the parameters measured were significantly (*p* > 0.05) affected by concentration and frequency of administration of *Lagenaria breviflora* fruit aqueous extract at the starter phase.

**Table 8:** Interactive effects of concentration and frequency of administration of *Lagenaria breviflora* fruit aqueous extract on behaviour of broiler chickens at the starter phase.

	**300 g/L *Lagenaria breviflora***			**500 g/L *Lagenaria breviflora***				
Parameters	0 time/week	3 times/week	4 times/week	0 time/week	3 times/week	4 times/week	SEM	*P*-value
***Feeding (freq.)***	17.88	17.46	22.29	20.88	19.49	19.66	1.22	0.084
***Drinking (freq.)***	16.78	22.02	18.99	19.87	19.87	16.83	2.08	0.231
***Dust bathing (freq.)***	0.77	0.99	0.98	0.71	0.98	0.98	0.19	0.987
***Wing flapping (freq.)***	4.98	5.91	10.99	5.13	5.30	5.76	1.28	0.117
***Walking (freq.)***	6.67	6.75	7.33	5.34	5.36	6.70	0.66	0.819

SEM, Standard error of mean.

### Main effects of concentration and frequency of administration of *Lagenaria breviflora* fruit aqueous extract on the behaviour of broiler chickens at the finisher phase

Table 9 presents the main effects of concentration and frequency of *Lagenaria breviflora* fruit aqueous extract administration on the behaviour of broiler chickens during the finisher phase. All parameters measured were significantly influenced (*p* < 0.05) by the mean effect of different concentrations of *Lagenaria breviflora* fruit aqueous extract. Broilers administered 300 g/L of *Lagenaria breviflora* fruit aqueous extract exhibited significantly higher feeding activity (26.05) compared to those given 500 g/L (19.50) of *Lageneria breviflora*. Higher drinking frequency was observed in the 300 g/L group (24.53 freq.) than in the 500 g/L group (20.28 freq.). A 300 g/L concentration led to increased dust-bathing behaviour (3.92 freq.) relative to the 500 g/L group (2.20 freq.). Birds that received 300 g/L showed more wing flapping (4.43 freq.) than those on 500 g/L (2.66 freq.). The 300 g/L group walked more frequently (7.38 freq.) compared to the 500 g/L group (4.60 freq.).

**Table 9:** Main effects of concentration and frequency of administration of *Lagenaria breviflora* fruit aqueous extract on behaviour of broiler chickens at finisher phase.

	Concentration				Frequency of administration				
Parameters	300 g/L	500 g/L	SEM	*P*-value	0 time/week	3 times/week	4 times/week	SEM	*P*-value
***Feeding***	26.05^a^	19.50^b^	0.83	0.000	23.99^a^	24.23^a^	20.11^b^	1.02	0.008
***Drinking***	24.53^b^	20.28^a^	0.95	0.002	22.40^ab^	25.99^a^	18.82^b^	1.16	0.000
***Dust bathing***	3.92^a^	2.20^ab^	0.51	0.019	2.85	4.16	2.18	0.62	0.078
***Wing flapping***	4.43^a^	2.66^b^	0.34	0.000	4.12^a^	3.81^ab^	2.69^b^	0.42	0.046
***Walking***	7.38^a^	4.60^b^	0.57	0.001	6.60^a^	6.90^ab^	4.47^b^	0.67	0.025

abc Means on the same row having different superscripts are significantly (*p* < 0.05) different.

SEM, Standard error of mean.

The frequency of administration of *Lagenaria breviflora* fruit aqueous extract significantly (*p* < 0.05) affected all parameters except dust bathing. Broilers without *Lagenaria breviflora* fruit aqueous extract (0/week) and those administered 3 times/week had higher feeding activities (23.99 and 24.23, respectively) than those receiving it 4 times/week (20.11). The highest drinking activity was observed in birds administered *Lagenaria breviflora* fruit aqueous extract 3 times/week (25.99), followed by those not receiving it (22.40), and the lowest in the 4 times/week group (18.82). Birds not receiving the extract exhibited more wing flapping (4.12) compared to those administered 4 times/week (2.69). The highest walking activity was observed in the non-administered group (6.60), with the lowest in the 4 times/week group (4.47).

### Interactive effects of concentration and frequency of administration of *Lagenaria breviflora* fruit aqueous extract on the behaviour of broiler chickens at the finisher phase

The interactive effects of concentration and frequency of administration of *Lagenaria breviflora* fruit aqueous extract on the behaviour of broiler chickens at the finisher phase are shown in Table 10.

**Table 10:** Interactive effects of concentration and frequency of administration of *Lagenaria breviflora* fruit aqueous extract on behaviour of broiler chickens at finisher phase.

	**300 g/L *Lagenaria breviflora***			**500 g/L *Lagenaria breviflora***				
Parameters	0 time/week	3 times/week	4 times/week	0 time/week	3 times/week	4 times/week	SEM	*P*-value
***Feeding***	29.38^a^	28.16^a^	20.63^b^	18.60^b^	20.31^b^	19.60^b^	1.44	0.004
***Drinking***	27.32^ab^	29.32^a^	16.9^c^	17.48^c^	22.67^ab^	20.68^bc^	1.65	0.000
***Dust bathing***	3.60	5.70	2.47	2.09	2.62	1.89	0.88	0.356
***Wing flapping***	5.47^a^	5.08^ab^	2.73^bc^	2.77^bc^	2.54^c^	2.66^bc^	0.59	0.041
***Walking***	9.28^a^	8.71^ab^	4.16^c^	3.93^c^	5.09^bc^	4.78^ab^	0.95	0.008

abc Means on the same row having different superscripts are significantly (*p* < 0.05) different.

SEM, Standard error of mean.

All measured parameters were significantly affected (*p* < 0.05), except dust bathing. These values were significantly higher than those recorded in all other treatment groups (*p* < 0.05). Birds on administration of 300 g/L of *Lagenaria breviflora* fruit aqueous extract 3 times/week had the highest drinking behaviour (29.32), while those administered *Lagenaria breviflora* fruit aqueous extract 4 times/week had the lowest drinking behaviour. Highest wing flapping (5.47) was observed in birds in the 0 time/week group administered 300 g/L *Lagenaria breviflora* fruit aqueous extract, while those on administration of 500 g/L of *Lagenaria breviflora* fruit aqueous extract had the lowest (2.54). Similar walking behaviour (4.16, 3.93) was observed in birds administered 300 g/L of *Lagenaria breviflora* fruit aqueous extract 4 times/week and those in the 0 time/week treatment group that received 500 g/L. These values were significantly lower than walking behaviour (9.28) observed in the control group receiving 300 g/L of *Lageneria breviflora* fruit aqueous extract 0 time/week (*p* < 0.05).

## Discussion

Phytobiotics exert a substantial and sustained beneficial impact on animal production and health. It is regarded as an effective substitute for synthetic antibiotics because it is natural, safe for both animals and humans, convenient to use and store, and can yield a return on investment. It also enhances the organism’s antioxidant status by optimising dietary quality and quantity. Results obtained revealed that *Lagenaria breviflora* fruit aqueous extract used in the present study significantly influenced the growth performance indices of the birds. Feed and water intake were reduced in birds administered aqueous extracts of *Lagenaria breviflora* fruit at varying concentrations. These parameters were also reduced as the frequency of administration increased. The observed reductions in feed and water intake among birds administered *Lagenaria breviflora* fruit aqueous extract, particularly at higher concentrations and more frequent dosing, may be attributed to the plant’s bioactive compounds such as tannins, saponins, and alkaloids. The reduction in feed and water intake observed in treated birds may be attributed to the bitter taste of phytochemicals such as tannins and saponins, which could reduce palatability. Furthermore, frequent exposure to these compounds may have induced gastrointestinal adaptations or mild satiety signalling, thereby suppressing appetite. This contrasts with the report by Toghyani et al. (2011), who observed that supplementation with certain herbal extracts did not significantly alter feed intake or growth performance in broiler chickens. Variations in response may be linked to differences in phytochemical type, dosage, or mode of administration, suggesting that the physiological effects of phytobiotics are plant- and concentration-dependent. According to Lee et al. (2004), broiler birds’ water intake is negatively impacted by the bitter-tasting essential oils. The significantly higher value obtained from birds fed 300 g/L, compared with the significantly lower value recorded from birds fed 500 g/L, may be due to the high concentration, which might have induced an off-flavour. The process of water metabolism in birds, which determines the water requirements and the levels of water intake in a broiler chicken, is a complex phenomenon (Steel et al. 1997) due to the fact that internal and external reactivity of water intake in birds is a stimulus governed by many factors, among which is the level of concentration of biochemical substances in water. Such substances in water can affect the taste buds of animals that consume water and can be readily altered by the concentration of chemical substances in the water (Nweze 2010). Additionally, Adeyemi et al. (2017) reported that the aqueous extract of *Lagenaria breviflora* fruit contains tannins and oxalates that may reduce water intake.

A reliable and fast means of assessing the clinical and nutritional health status of animals includes the use of blood analysis, because ingestion of bioactive components has measurable effects on blood composition (Maxwell et al. 1990; Olabanji et al. 2007) and may be considered as an appropriate measure of long-term nutritional status (Khan and Zafar, 2005). Haematological studies have been shown to be useful for disease prognosis, treatment, and monitoring feed stress (Togun and Oseni 2005). They are also good indicators of animal physiological health. *The aqueous extract of Lagenaria breviflora fruit had no significant effect* on the haematological parameters assessed in this study. The lack of a significant effect of aqueous extract of *Lagenaria breviflora* fruit on the haematological parameters assessed in this study may be attributed to several physiological and experimental factors. One plausible explanation is the development of tolerance in broiler chickens. Continuous exposure to the extract’s bioactive constituents may have led to an adaptive physiological response, thereby stabilising blood parameters over time. Additionally, the interaction between the different concentrations and administration frequencies used in this study may have resulted in a compensatory effect, neutralising potential haematological variations that might have otherwise been observed with single-dose regimens. Furthermore, the basal diet administered during the study was nutritionally balanced, potentially buffering or minimising any haematological influence of the extract. The birds’ health status and the minimal environmental stress they were subjected to may also have contributed to the stability of their blood indices, as haematological responses are often sensitive to stress and disease. Another contributing factor could be the variation in the phytochemical composition of the extract, which is influenced by factors such as fruit maturity, extraction method, and storage conditions. These variables can affect the bioavailability and potency of the active compounds responsible for physiological changes. This result contradicts the findings of Ekunseitan et al. (2017) and Nworgu et al. (2018), who reported significant effects on packed cell volume (PCV), red blood cells (RBC) and haemoglobin levels in birds administered *Lageneria breviflora* fruit aqueous extract. Additionally, this contradicts the study by which reported that lymphocyte and eosinophil counts differed significantly with increasing concentrations of *aqueous extract of Lagenaria breviflora fruit*.

However, the non-significant result observed in this study aligns with the findings of Al-Baghdadi (2011), who suggested that varying concentrations of water-soluble plant extracts in drinking water may exert differential effects on haematological indices. According to Bounous and Stedman (2000), the normal ranges of chicken haematological parameters are: 2.5–3.5 × 10^6^ µL (RBC), 22–35% (PCV), 7–13 g/dl (haemoglobin) and 12–30 × 10^3^ µL (WBC). All values obtained for these parameters in this study were within these reference ranges, which could indicate that the bird’s bone marrow is properly functioning, thereby stimulating RBC formation. The physiological relevance of these haematological parameters in broilers was maintained, as observed in previous studies in which non-significant changes did not adversely affect bird health (Al-Baghdadi 2011). These findings indicate that *the aqueous extract of Lagenaria breviflora fruit did not significantly alter haematological parameters*, reinforcing its potential safety in broiler production without causing adverse physiological effects.

The results indicate that both the concentration and the frequency of administration significantly affected serum biochemical indices in broiler chickens, with the 500 g/L concentration yielding higher total protein (52.31 g/L) and albumin (34.64 g/L) levels than the 300 g/L concentration. Although a slight increase in AST was observed at higher concentrations, the simultaneous elevation in HDL and marked reduction in malondialdehyde (MDA) levels support the extract’s potent antioxidant activity of *Lagenaria breviflora* (Orisakeye and Johnson 2015). Furthermore, increasing the frequency of administration from three to four times weekly was associated with lower serum glucose, reduced urea and creatinine levels, and a better lipid profile, as indicated by lower cholesterol and higher HDL concentrations, collectively suggesting improved metabolic regulation and potential renal protection. These findings imply that the bioactive phytochemicals in *the aqueous extract of Lagenaria breviflora fruit, such as flavonoids and tannins, confer benefits* by enhancing antioxidant defences and modulating protein and lipid metabolism, thereby offering a natural alternative to synthetic additives in poultry production (Tomori et al. 2007). Biochemical components are sensitive to toxic elements in feeds and plant extracts and are particularly valuable for monitoring toxicity, especially for feed constituents that affect blood formation (Bobulescu and Moe 2012). The results of serum biochemical indices measured in this study indicated that the frequency of administration of aqueous extract of *Lagenaria breviflora* fruit reduced urea concentration. This is in contrast to the results of Ekunseitan et al. (2017), who found that laying birds given *Lagenaria breviflora* fruit aqueous extract had significantly higher serum uric acid levels than birds administered the extract 0 times/week. The balance between urate reabsorption and resecretion determines renal uric acid excretion, and the kidney is the primary regulator of serum uric acid (Simoyi et al. 2002). Consequently, serum uric acid levels may be considered a therapeutic target for preventing thromboembolic events and could serve as a predictor of antithrombotic medication selection (Ţăpoi et al. 2021). The increase in AST observed in this study was concentration-dependent, with birds offered 500 g/L of *Lagenaria breviflora* fruit aqueous extract showing the highest significant value. Although AST levels increased at higher concentrations, these values remained within physiological ranges; however, this should be interpreted cautiously as elevated AST may indicate increased hepatic activity or mild stress (Altmann, 1979). AST enzyme activities in the serum are generally increased with muscle or hepatocellular damage. The higher serum AST activity observed in birds in the 500 g/L *Lagenaria breviflora* fruit aqueous extract group may be due to increased hepatic metabolic demand. This is consistent with the study by Balogun et al. (2014), who reported that high doses of *Lagenaria breviflora* fruit extract in rats led to increased serum AST levels, which the authors attributed to heightened metabolic demands on the liver and consequent cell membrane degeneration, resulting in enzyme leakage. In addition, AST was higher in birds administered *Lagenaria breviflora* fruit aqueous extract 0 times/week than in birds administered *Lagenaria breviflora* fruit aqueous extract thrice and four times a week. This is in line with the study by Ekunseitan et al. (2017), who observed that birds not receiving the extract (ie 0 times/week) had higher AST levels than those administered the extract three or four times per week, suggesting that regular administration may help maintain liver integrity.

Plant extracts have been reported previously by Ţăpoi et al. (2021) to contain certain compounds that reduce hepatic damage. These plants are a rich source of alkaloids, flavonoids, diterpenoid, tannins, lipids, sterols etc. with anti-microbial and hepatic protective effects Alagbe et al. (2020) . Serum creatinine, a chemical waste product of muscle metabolism, is a simple measure of renal function because the kidney maintains blood creatinine within the normal range Salama et al. (2024). Results from the current study showed that the frequency of administration of aqueous extract of *Lagenaria breviflora* fruit reduced creatinine levels compared with the control group. This showed that the administration of *aqueous extract of Lagenaria breviflora fruit did not adversely affect* the kidneys of chickens. Reduced malondialdehyde (MDA) levels were observed in birds following oral administration of a 500 g/L aqueous extract of *Lagenaria breviflora* fruit, suggesting a potential role in mitigating oxidative stress. This observation may be attributed to Lagenaria’s appreciable antioxidant activity (Erasto and Mbwambo, 2009), which enhances and stabilises the cell membrane by activating antioxidants and preventing excess free radicals in the system. The observed reduction in malondialdehyde levels suggests enhanced antioxidant defence, likely mediated by flavonoids and phenolic compounds present in the extract. Studies have shown that natural herbal products contain various phytochemicals and possess potent antioxidant activity (Liu et al. 2008; Orlowski et al. 2018). Research by Ekunseitan (2017) also found that supplementation with an aqueous extract of *Lagenaria breviflora* fruit at 150 g/L significantly reduced liver MDA levels, thereby improving oxidative stability in the birds.

Results obtained in this study reveal that the behaviour indices of the birds at the finisher phase were improved at a lower concentration of *Lagenaria breviflora* fruit aqueous extract. This was demonstrated by the birds’ expression of comfort behaviours, such as dust bathing, at this concentration, suggesting that the birds are more comfortable at this level. Comfort activities are carried out when fundamental requirements are met (Duncan and Mench, 1993). Natural behaviour, such as dust bathing, has been found to help prevent frustration Cindy et al. (2011). According to Shields et al. (2004), the movement pattern associated with dust bathing may aid in strengthening leg muscles and reducing walking disabilities and leg deformities.

In conclusion, this study has demonstrated that aqueous extract of *Lagenaria breviflora* fruit can be effectively utilised as a natural phytogenic supplement in broiler chicken production. The concentration and frequency of administration of *Lagenaria breviflora* fruit aqueous extract did not influence the haematological parameters of broiler chickens but, it had significant effects on the serum biochemical indices of the birds. At the starter phase, the behaviours of the birds were not affected by the concentration and frequency of administration of *Lagenaria breviflora* fruit aqueous extract. However, the observed modifications in behavioural patterns at the finisher phase suggest a potential role of the extract in promoting bird comfort and welfare. The effect of administration of the extract at different concentration and frequency was not consistent on the growth performance parameters of the chickens. However, further studies are recommended to elucidate the underlying bioactive compounds, mechanisms of action, and long-term effects under different environmental and management conditions.

## Conclusions

The aqueous macerate of *Lagenaria breviflora* influenced behavioural activity and improved selected biochemical indices, particularly antioxidant status and may offer potential benefits in poultry production. However, it did not enhance growth performance and was associated with reduced feed intake at higher concentrations and frequencies.

Therefore, while the extract shows potential as a phytogenic additive, its application should be approached cautiously, and further studies are required to optimise dosage and standardise its composition.

The results of this study demonstrate that oral administration of the aqueous extract of *Lagenaria breviflora* fruit influenced broiler chickens primarily by modulating feed and water intake, behavioural activity, and serum biochemical indices, rather than by enhancing growth performance. Higher extract concentration (500 g/L) and increased administration frequency reduced feed and water intake, likely contributing to the observed decreases in body weight gain and final body weight relative to the control group. This suggests that the extract may negatively affect growth performance, possibly due to reduced palatability or the presence of bioactive compounds such as tannins and saponins. The extract did not adversely affect the general physiological or health status of the birds. However, it modulated serum biochemical indices, as evidenced by increased total protein, albumin, and high-density lipoprotein levels, alongside reduced malondialdehyde levels, suggesting improved antioxidant status and metabolic activity, particularly at the higher concentration. Behavioural responses were not affected during the starter phase, but during the finisher phase, a lower extract concentration (300 g/L) was associated with increased expression of activity-related behaviours such as feeding, drinking, walking, and wing-flapping, indicating improved behavioural responsiveness and possible welfare benefits at this level.

Overall, while the aqueous extract of *Lagenaria breviflora* fruit may contribute to enhanced physiological stability and antioxidant capacity, its lack of positive effect and potential negative impact on growth performance suggest that it should not be considered a growth-promoting additive under the conditions of this study. Further research is required to optimise dosage, improve extract standardisation, and better understand the mechanisms underlying its biological effects.
